# On the modelling of M_2_ tidal magnetic signatures: effects of physical approximations and numerical resolution

**DOI:** 10.1186/s40623-018-0967-5

**Published:** 2018-12-11

**Authors:** Jakub Velímský, Alexander Grayver, Alexey Kuvshinov, Libor Šachl

**Affiliations:** 1 Department of Geophysics, Faculty of Mathematics and Physics, Charles University, V Holešovičkách 2, 180 00 Prague, Czech Republic; 2 Institute of Geophysics, ETH Zurich, Sonneggstrasse 5, 8092 Zurich, Switzerland

**Keywords:** Electromagnetic induction, Ocean tides, Ocean-mantle electromagnetic coupling

## Abstract

The magnetic signatures of ocean $\hbox {M}_{2}$ tides have been successfully detected by the low-orbit satellite missions CHAMP and Swarm. They have been also used to constrain the electrical conductivity in the uppermost regions of the Earth’s mantle. Here, we concentrate on the problem of accurate numerical modelling of tidally induced magnetic field, using two different three-dimensional approaches: the contraction integral equation method and the spherical harmonic-finite element method. In particular, we discuss the effects of numerical resolution, self-induction, the galvanic and inductive coupling between the oceans and the underlying mantle. We also study the applicability of a simplified two-dimensional approximation, where the ocean is approximated by a single layer with vertically averaged conductivity and tidal forcing. We demonstrate that the two-dimensional approach is sufficient to predict the large-scale tidal signals observable on the satellite altitude. However, for accurate predictions of $\hbox {M}_{2}$ tidal signals in the areas with significant variations of bathymetry, and close to the coastlines, full three-dimensional calculations are required. The ocean–mantle electromagnetic coupling has to be treated in the full complexity, including the toroidal magnetic field generated by the vertical currents flowing from and into the mantle.
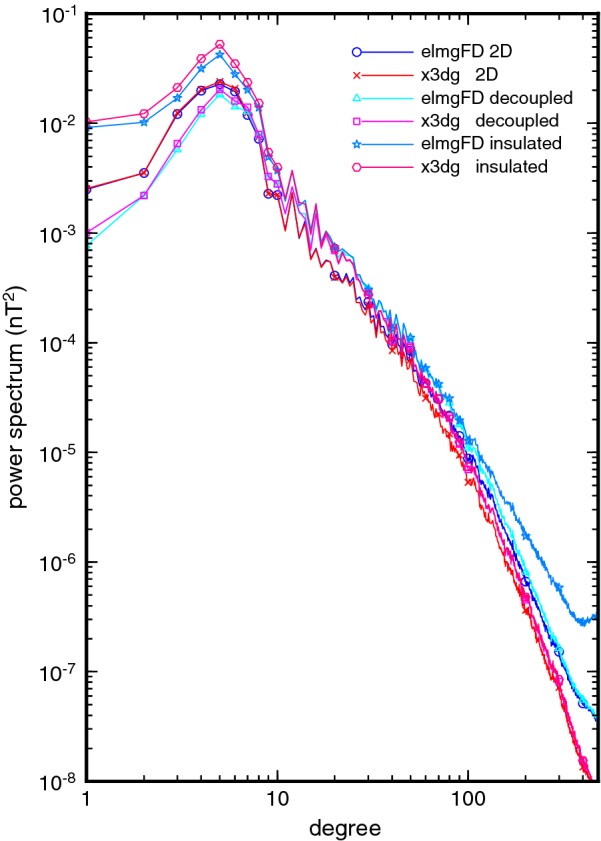

## Introduction

The phenomenon of electromagnetic fields induced in the Earth’s oceans by the motion of the conductive seawater in the presence of the main magnetic field has been known for decades (Sanford 1971). In particular, the electromagnetic signatures of the ocean flows driven by the gravitational forcing of the Sun and the Moon have recently attracted an increased interest in the geomagnetic field community. Although the existence of compound tides generated by nonlinear ocean dynamics is also well established (Einšpigel and Martinec 2017), the fundamental part of tidal flows consists of the constituents present at discrete frequencies related to the movements of the celestial bodies. This attribute allows us to distinguish the tidally induced magnetic field from the magnetic field originated in the Earth’s liquid core, in the lithosphere, or the external contributions from the magnetosphere and ionosphere, and their induced counterparts. Indeed, the magnetic signatures of the principal lunar semi-diurnal constituent $\hbox {M}_{2}$, the larger lunar elliptic semi-diurnal constituent $\hbox {N}_{2}$, and partially also the lunar diurnal constituent $\hbox {O}_{1}$ have been detected in ground geomagnetic observatory data (Maus and Kuvshinov 2004) and seafloor measurements in the Northwestern Pacific (Schnepf et al. 2014). Similarly, the low-orbit satellite missions, such as CHAMP (Sabaka et al. 2015; Tyler et al. 2003) and Swarm (Sabaka et al. 2016), have successfully recovered the $\hbox {M}_{2}$ and partially also the $\hbox {N}_{2}$ tidally induced magnetic signals. Other tidal signal, such as $\hbox {S}_{2}$, $\hbox {K}_{1}$, and $\hbox {P}_{1}$ can be obscured by external fields (Guzavina et al. 2018; Maus and Kuvshinov 2004). Consequently, the tidal signals extracted from satellite data were used to constrain the electrical conductivity across the lithosphere–asthenosphere boundary (Grayver et al. 2016, 2017). Recently, Schnepf et al. (2018) have compared the numerical predictions of magnetic fields generated by oceanic and ionospheric tides. The idea to assimilate the magnetic field measurements directly into the global ocean flow modelling has been implemented by Irrgang et al. (2017); however, tides were not considered there. Another application of the forward modelling of tidal signals is the construction of base functions optimized for tidal signal recovery from satellite and observatory data (Telschow et al. 2018).

The question of accurate calculation of the tidal magnetic signatures embraces several problems. The choice of the tidal flow model from a plethora of purely hydrodynamical or assimilative approaches definitely plays a role (Saynisch et al. 2018). The effect of the seawater conductivity and its seasonal variations has been discussed by Saynisch et al. (2016). This paper returns back to the basic physical formulation of the problem of tidally induced magnetic fields. The two-dimensional (2-D) horizontal characteristics of the tidal flows were traditionally exploited in the solution of the electromagnetic induction equation by assuming a 2-D conductive sheet of infinitesimal or finite thickness with a prescribed distribution of 2-D imposed surface electric currents. Here, we refer to a recent detailed derivation by Tyler (2017) and references therein. Another level of simplification is related to the treatment of the underlying solid mantle. In the simplest case, it is assumed to be a perfect insulator; more advanced methods allow for purely inductive, or inductive and galvanic coupling between the mantle and the ocean. Our paper aims to study the applicability of such simplifications using two state-of-the-art, fully three-dimensional (3-D) global modelling methods. Although the 3-D codes have been around for some time, only recently it has become computationally feasible to carry out such an analysis at sufficient resolutions. We do not attempt to play down the computational effectiveness of the approximate solutions. As discussed later in this manuscript, the computational costs of the fully 3-D solutions at high resolution are still prohibitive for inverse studies. Here, we merely test the validity of various physical approximations for modelling the magnetic field at the Earth’s surface and low-orbit satellite altitude induced by the principal lunar semi-diurnal constituent $\hbox {M}_{2}$.

The paper is organized as follows. We begin by recalling the 3-D formulation of the problem stemming from the quasi-stationary Maxwell equations and proceed to a 2-D approximation. We then look at the effects of electromagnetic interaction of the ocean and mantle, treating separately the galvanic and inductive coupling. Using two independent solutions ensures that the observed effects correspond to real physical phenomena and issues of implementation, resolution, and numerical accuracy are clearly separated.

## Tidally induced magnetic field in the Earth’s oceans

### The three-dimensional electromagnetic induction equation

The magnetic field $\varvec{B}(\varvec{r};t)$ and the electric field $\varvec{E}(\varvec{r};t)$ induced in the Earth’s oceans by the motion of the saltwater in the presence of the Earth’s main magnetic field $\varvec{B}_{\mathrm{M}}(\varvec{r};t)$ are governed by the quasi-static Maxwell equations,

1 $$\begin{aligned} \nabla \times \varvec{B}=\, & {} \mu _0\left( \varvec{j}+ \varvec{j}^{\mathrm{imp}}\right) , \end{aligned}$$

2 $$\begin{aligned} \nabla \times \varvec{E}=\, & {} -\frac{\partial \varvec{B}}{\partial t}, \end{aligned}$$

3 $$\begin{aligned} \nabla \cdot \varvec{B}=\, & {} 0. \end{aligned}$$

Here, $\mu _0$ is the magnetic permeability of vacuum. The electric current density $\varvec{j}(\varvec{r};t)$ is related to the electric field through the Ohm’s law,

4 $$\begin{aligned} \varvec{j}= \sigma \varvec{E}, \end{aligned}$$

with $\sigma (\varvec{r})$ representing the electrical conductivity, in general both laterally and radially varying. The imposed electric current density $\varvec{j}^{\mathrm{imp}}(\varvec{r};t)$ and the imposed electric field $\varvec{E}^{\mathrm{imp}}(\varvec{r};t)$ are defined as

5 $$\begin{aligned} \varvec{j}^{\mathrm{imp}}= \sigma \varvec{E}^{\mathrm{imp}}= \sigma \left( \varvec{u}\times \varvec{B}_{\mathrm{M}}\right) . \end{aligned}$$

Here, $\varvec{u}(\varvec{r};t)$ is the spatially and temporally dependent velocity of the ocean flow. In general, it is fully three-dimensional (3-D), containing all three vector components varying along all three dimensions in the oceans. The same equations with zero imposed electric field or current also hold in the conductive Earth’s mantle. We have implicitly assumed that $\varvec{B}_{\mathrm{M}}$ is a potential field, its amplitude is much larger than that of the induced field, and its time variations are too slow to be considered in the diffusion process. At the Earth surface, $r=a$, the induced magnetic field is coupled to a scalar magnetic potential $U(\varvec{r};t)$,

6 $$\begin{aligned} \varvec{B}= -\nabla U\quad \hbox {at}~{r=a}, \end{aligned}$$

that satisfies the Laplace equation in the insulating atmosphere, and assuming the absence of external sources, it disappears as $r\rightarrow \infty$.

Since the tidal flows are dominated by signals at discrete frequencies, it is common to reformulate the problem in the frequency domain. Assuming the $\exp (- \hbox{i} \omega t)$ dependence of the velocities and induced fields, and ignoring the notation distinction between time-domain and frequency-domain variables, we write

7 $$\begin{aligned} \nabla \times \varvec{B}=\, & {} \mu _0\left( \varvec{j}+ \varvec{j}^{\mathrm{imp}}\right) , \end{aligned}$$

8 $$\begin{aligned} \nabla \times \varvec{E}=\, & {} \hbox {i}\omega \varvec{B}, \end{aligned}$$

9 $$\begin{aligned} \nabla \cdot \varvec{B}=\, & {} 0. \end{aligned}$$

In this paper, we consider only the principal lunar semi-diurnal constituent $\hbox {M}_{2}$ with a period of 12.42 h, and thus angular frequency $\omega =1.4\times 10^{-4}{\hbox {rad}}/{\hbox {s}}$. This component has by far the largest magnetic signatures, which have been reliably detected by satellite observations (Sabaka et al. 2016).

The Ampère and the Faraday laws (7–8) can be combined with Eqs. (4) and (5) into a single second-order electromagnetic induction (EMI) equation for the magnetic field vector $\varvec{B}$,

10 $$\begin{aligned} \nabla \times \left( \frac{1}{\sigma }\nabla \times \varvec{B}\right) - \hbox {i}\omega \mu _0\varvec{B}= \mu _0\nabla \times \varvec{E}^{\mathrm{imp}}. \end{aligned}$$

Because of linearity, the induction caused by external sources in the ionosphere and magnetosphere represents an independent solution, which is not considered here, and we refer to a comprehensive benchmark of externally induced magnetic fields by Kelbert et al. (2014).

The  solution of Eqs. (7–8) or (10) in the computational domain comprising the oceans and the solid Earth below is a challenging task, in particular in the presence of large lateral variations of conductivity, which coincide with the spatial distribution of the source term on the right-hand side. Here, we employ two numerical methods.

The elmgFD code uses the spherical harmonic-finite element approach, introduced by Martinec (1999), to solve the EMI Eq. (10). It has been recently rewritten using modern parallelized FFT and LAPACK libraries, the BiCGStab(2) iterative solver (Sleijpen and Fokkema 1993), applying an effective pre-conditioner based on the spherical harmonic solution of the 1-D problem (Martinec 1999), and incorporating the zero external field boundary condition (6) at the Earth–atmosphere interface (Velímský and Martinec 2005), and the internal forcing (5). The lateral resolution is controlled by the maximum spherical harmonic degree $j_{\mathrm{max}}$. The code is very effective in terms of memory usage, avoiding the storage of the full problem matrix. At the highest resolution employed here, $j_{\mathrm{max}}=480$ with 102 3-D layers in the oceans, and additional 100 1-D layers in the mantle, the calculation of one forward run required 30 GiB of memory and took about 2 days on a 12-core modern PC. Note that the memory requirements scale linearly with the total number of layers and quadratically with $j_{\mathrm{max}}$. The duration of a single iteration scales with the third power of $j_{\mathrm{max}}$ and linearly with the number of 3-D layers. Obviously, the total number of iterations depends on the requested accuracy, and convergence rate can be influenced also by the range of lateral conductivity variations. In general, the presented results are at the edge of practicality for single-frequency forward calculations. When employed in the inverse modelling, significant reduction of lateral and radial resolution is needed. For accurate transformations between the spatial and spherical harmonic domains by means of the Gauss–Legendre quadrature, the electrical conductivity, the tidal flows, and the solution itself are distributed on an irregular grid in colatitude, and bilinear interpolation is used for conversions to and from a regular grid.

The x3dg (Kuvshinov 2008) code is based on the contracting integral equation (CIE) approach (Pankratov et al. 1995; Singer 1995). Within the approach, the Maxwell Eqs. (7, 8) are transformed to CIE which is solved using Krylov subspace iterations with pre-calculated Green tensors for a 1-D medium. The approach allows for computing the EM fields in the Earth’s models with fully 3-D conductivity distributions. One of the advantages of the x3dg method lies in the fast iterative process with guaranteed convergence, as the condition number of the system matrix depends only on the square root of maximum lateral conductivity contrast. On the other hand, it is partially offset by the large memory requirements of the Green tensors in the current implementation and lack of parallelization for single-frequency calculations. For example, using a single ocean layer at $0.25^\circ$ lateral resolution requires over 100 GiB of memory or swap disc space with corresponding speed penalty. The memory requirements scale quadratically with the number of 3-D layers and latitudinal resolution, quickly saturating even the most advanced shared-memory architecture available today.

### The three-dimensional and the two-dimensional approaches

**Fig. 1:**
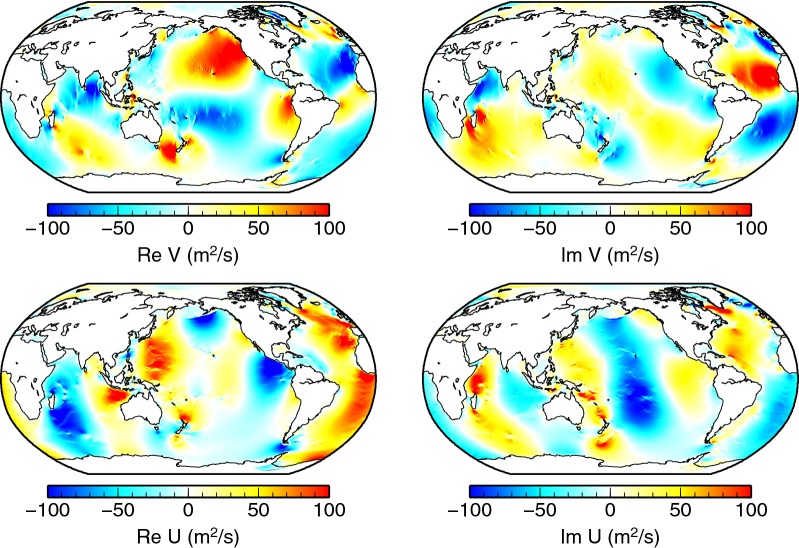
$\hbox {M}_{2}$ transports. The northward component *V* and the eastward component *U* of the $\hbox {M}_{2}$ transports according to the TPXO8-atlas model (Egbert and Erofeeva 2002)

The gravitational forcing that drives the tidal flows is almost independent of the vertical coordinate in the ocean, and therefore, the ocean tides are usually modelled in the two-dimensional (2-D) barotropic approximation (Hendershott 1973). The full velocity field $\varvec{u}(\varvec{r})$ for a given tidal constituent is not calculated, and only the horizontal transport is available. It is defined as a vertical integral of the horizontal velocity $\varvec{u}_{\mathrm{H}}$,

11 $$\begin{aligned} \varvec{U}(\vartheta ,\varphi ) = \int \limits _{a-b(\vartheta ,\varphi )}^{a} \varvec{u}_{\mathrm{H}}(r,\vartheta ,\varphi ){\mathrm{d}}r, \end{aligned}$$

where $b(\vartheta ,\varphi )$ represents the local bathymetry at colatitude and longitude $(\vartheta ,\varphi )$. The small contribution associated with laterally varying surface elevation is neglected in Eq. (11). Figure 1 shows the $\hbox {M}_{2}$ transport from an assimilative barotropic model TPXO8-atlas (Egbert and Erofeeva 2002) that has been used in this study.

The vertical velocity component stemming from the baroclinic internal tides (Kantha and Tierney 1997) is not considered here. A reliable baroclinic tidal model with assimilated altimetry data has not been published yet. Besides, the frequency shift and spread of baroclinic tides would require a multi-frequency or time-domain approach to the solution of the induction Eq. (10), a task out of the scope of the present paper.

In order to calculate the tidally induced magnetic fields in the 3-D settings, we need to specify the distribution of electrical conductivity in the uppermost layers, comprising the oceans, the continents, and the underlying crust, $\sigma _{\mathrm{3D}}(r,\vartheta ,\varphi )$. In the present paper, we start with a simplified approach, based on only two values of electrical conductivity: one for the seawater and one for the solid crust,

12 $$\begin{aligned} \sigma _{\mathrm{3D}}(r,\vartheta ,\varphi ) = \left\{ \begin{array}{ll} \sigma _{\mathrm{ocean}}= 3.2 {\hbox {S}}/{\hbox {m}} &\quad \hbox {for}\quad r\ge a-b(\vartheta ,\varphi ),\\ \sigma _{\mathrm{crust}}= 0.001 {\hbox {S}}/{\hbox {m}}&\quad \hbox {for}\quad a-h \le r< a-b(\vartheta ,\varphi ), \end{array}\right. \end{aligned}$$

with $b(\vartheta ,\varphi )=0$ at the continents. Here, $h=8000~\hbox {km}$ is the ocean layer thickness, which should be larger than the maximum bathymetry. The actual value of $\sigma _{\mathrm{crust}}$ has only small influence on the tidally induced magnetic fields, spatially limited to coastal areas.

**Fig. 2:**
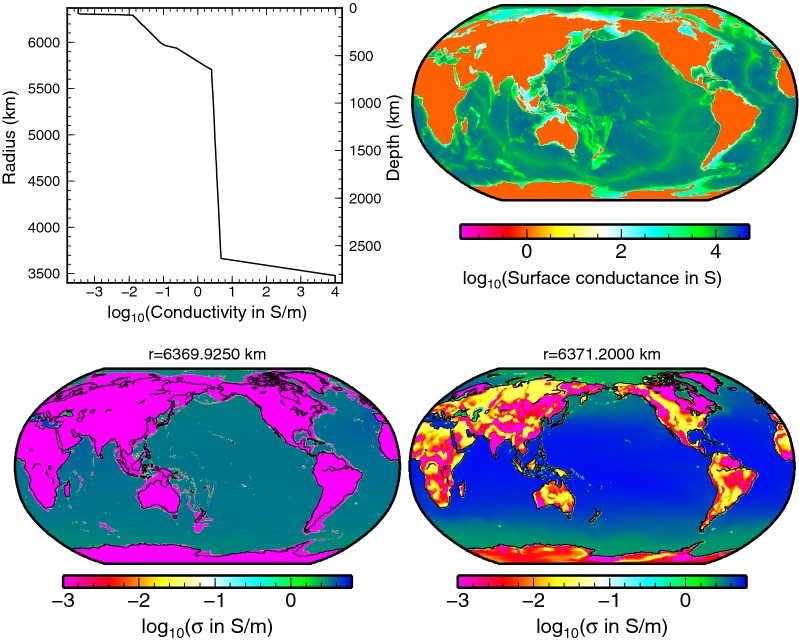
Electrical conductivity. Top left: The 1-D electrical conductivity profile of the Earth’s mantle after Grayver et al. (2017). Top right: Map of the electrical surface conductance of the oceans and continents used in the 2-D models. Bottom: Electrical conductivity map including the effects of seawater salinity, temperature, and the presence of sediments. Cross sections at 1.275 km depth (left) and at the surface (right)

An alternative approach introduces a 3-D electrical conductivity distribution $\sigma _{\mathrm{3D}}^{\mathrm {(T,s)}}$ which is based on the collocated seawater temperature and salinity (*T*, *s*) measurements (Tyler et al. 2017). Variable thickness of ocean, continental, and shelf sediments is also incorporated along the lines presented by Everett et al. (2003). Two cross sections of $\sigma _{\mathrm{3D}}^{\mathrm {(T,s)}}$ are shown in the bottom panels of Fig. 2. Near the surface, the seawater conductivity shows significant variations with colatitude. In the deep oceans, the lateral variations are suppressed.

In order to construct the 3-D structure of the imposed electric currents or electric fields, we assume that the horizontal ocean velocity is constant within the entire ocean column. Then, we obtain from (11),

13 $$\begin{aligned} \varvec{u}_{\mathrm {3D}}(r,\vartheta ,\varphi ) = \frac{\varvec{U}(\vartheta ,\varphi )}{b(\vartheta ,\varphi )}, \end{aligned}$$

for $a-b(\vartheta ,\varphi )\le r \le a$, and zero elsewhere. The horizontal transport (11) is conserved, and Eq. (5) can be used to calculate the 3-D imposed electric field or electric current. While the velocity field calculated by Eq. (13) has only the horizontal components, its spatial distribution follows the bathymetry profile.

In the two-dimensional approach, the radial conductivity profile is averaged and replaced with a scaled, two-dimensional conductivity map

14 $$\begin{aligned} \sigma _{\mathrm{2D}}(\vartheta ,\varphi ) = \frac{1}{h} \int \limits _{a-h}^{a}\sigma _{\mathrm{3D}}(r,\vartheta ,\varphi ){\mathrm {d}}r. \end{aligned}$$

Similarly, the imposed currents are also vertically integrated,

15 $$\begin{aligned} \varvec{j}^{\mathrm{imp}}_{\mathrm{2D}}(\vartheta ,\varphi )= & {} \frac{1}{h} \int \limits _{a-b(\vartheta ,\varphi )}^{a} \sigma _{\mathrm{3D}}(r,\vartheta ,\varphi ) \varvec{u}_{{\mathrm {H}}}(r,\vartheta ,\varphi ) \times \varvec{B}_{\mathrm{M}}(r,\vartheta ,\varphi ){\mathrm {d}}r \nonumber \\= & {} \frac{1}{h}\sigma _{\mathrm{ocean}}\varvec{U}(\vartheta ,\varphi )\times \varvec{B}_{\mathrm{M}}(a,\vartheta ,\varphi ), \end{aligned}$$

under the assumption that the radial variability of the main field across the ocean is negligible, $\varvec{B}_{\mathrm{M}}(r,\vartheta ,\varphi )\approx \varvec{B}_{\mathrm{M}}(a,\vartheta ,\varphi )$. The imposed electric fields are then expressed as

16 $$\begin{aligned} \varvec{E}^{\mathrm{imp}}_{\mathrm{2D}}(\vartheta ,\varphi ) =\frac{\varvec{j}^{\mathrm{imp}}_{\mathrm{2D}}(\vartheta ,\varphi )}{\sigma _{\mathrm{2D}}(\vartheta ,\varphi )}. \end{aligned}$$

Note that the calculation of $\varvec{E}^{\mathrm{imp}}_{\mathrm{2D}}$ via Eq. (16) assures consistency between the Maxwell Eqs. (7–8) and the EMI Eq. (10). On the other hand, setting simply $\varvec{E}^{\mathrm{imp}}_{\mathrm{2D}}= \varvec{U}\times \varvec{B}_{\mathrm{M}}/ h$ does not.

The 2-D approach presented here only suppresses the radial variations of conductivity and imposed currents in the oceans. The formulation still includes both the galvanic and inductive coupling with the underlying mantle. This is in contrast with the 2-D approach by Tyler (2017), where the galvanic coupling is omitted.

### Inductive and galvanic interaction with the underlying mantle

One of the goals of this study is to assess the importance of both inductive and galvanic coupling of the ocean magnetic field with the underlying conductive mantle. We use a recent global 1-D electrical conductivity model by Grayver et al. (2017), derived from Swarm and CHAMP satellite data using a combination of magnetospheric and tidal forcing. The profile, as shown in the upper left panel of Fig. 2, features an abrupt increase in electrical conductivity across the lithosphere–astenosphere boundary. The conductivity further increases in the transition zone in the upper mantle. A highly conductive core is also included in the model.

Two models with limited physics are calculated by both numerical methods to study the ocean–mantle interactions. In the so-called *decoupled* model, the galvanic coupling is removed. In x3dg, it is achieved by inserting a 100-m thin layer with electrical conductivity of $10^{-12}{\hbox {S}}/{\hbox {m}}$ between the ocean and the mantle. The spherical harmonic approach elmgFD allows us to directly disable the toroidal magnetic field and hence any radial electric currents. Only the inductive interaction between the oceans and the mantle is preserved.

The second model, marked as *insulated* in the following figures and discussion, assumes an insulating mantle below the ocean. In elmgFD, a perfectly insulating analytical boundary condition is applied at $r=a-h$, while extremely low conductivity of $10^{-12}{\hbox {S}}/{\hbox {m}}$ is used in x3dg both for the mantle and the core. Hence, the effect of mantle conductivity is completely suppressed.

## Results

### Effect of lateral resolution and dimensionality

In the first series of runs, we have calculated the magnetic signatures of the TPXO8-atlas $\hbox {M}_{2}$ tides using the elmgFD and x3dg codes with the 2-D settings and full physics. The electrical conductivity was assembled according to Eq. (14), and the elmgFD and x3dg solutions were, respectively, forced by the imposed electric field (16) or electric current (15). The spherical harmonic truncation degree of elmgFD was set in turn to 120, 240, and 480. Similarly, the lateral resolution of x3dg was increased from $1^\circ \times 1^\circ$ to $0.5^\circ \times 0.5^\circ$ and finally to $0.25^\circ \times 0.25^\circ$. In addition, the 3-D solutions were calculated by elmgFD at the highest lateral resolution and discretizing the ocean radially into 40 and 102 layers, respectively, for the conductivity models $\sigma _{\mathrm{3D}}$ and $\sigma _{\mathrm{3D}}^{\mathrm {(T,s)}}$. The imposed electric field was calculated using Eq. (5) with quasi-3-D flows assembled according to Eq. (13). The large memory demands of x3dg have so far prevented us from calculating such high-resolution 3-D solutions with this code.

**Fig. 3:**
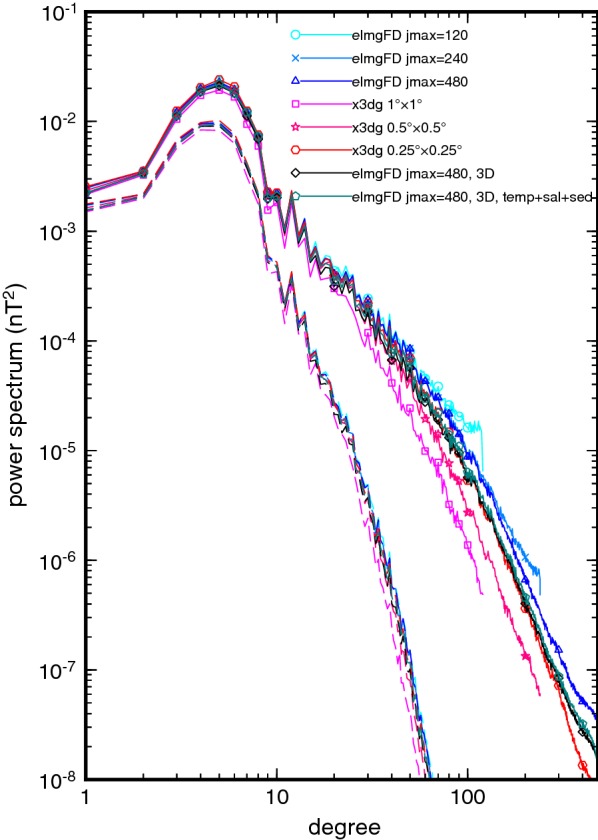
Geomagnetic power spectra. Geomagnetic power spectra at the Earth surface (solid lines and symbols) and at the satellite altitude of 400 km (dashed lines). Results of the 2-D approaches elmgFD and x3dg are shown for increasing resolution and compared with two calculations of elmgFD in 3-D settings

Figure 3 shows the power spectra of the tidally induced field at the Earth’s surface, and at the altitude of 400 km, typical for low-orbit satellite missions such as Swarm. The spectra were calculated using the formula by Maus (2008, eq. 21), taking into account the increasing number of coefficients with the spherical harmonic degree. The maximum average power is present at degree five. The spectra follow the power law for degrees above 10 without reaching a plateau. That suggests that the induced field remains correlated across different spatial scales. At the lowest resolution, we can observe significant differences between the 2-D and 3-D methods, diverging for higher spherical harmonic degrees. An interesting observation is that the elmgFD method predicts systematically larger spectra than the x3dg method. As the lateral resolution increases, the differences between both methods are reduced. A possible explanation lies in the use of global base functions in elmgFD, compared to a local discretization applied in x3dg. Truncating the elmgFD solution at a lower degree prevents additional diffusion of magnetic field energy into higher degrees and hence increases the spectrum.

**Fig. 4:**
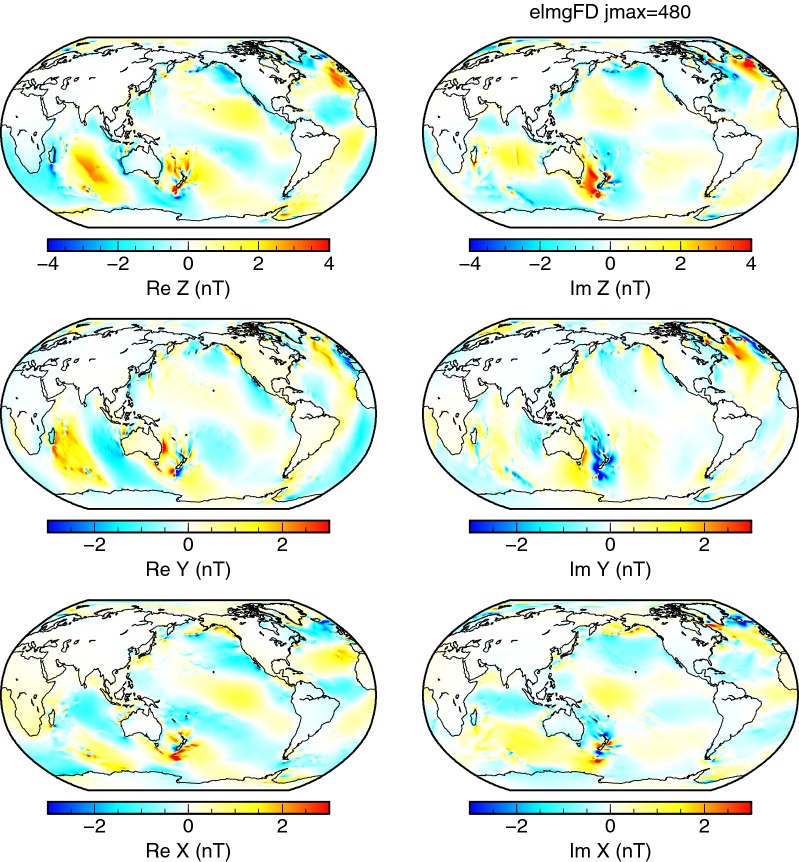
Magnetic signatures of $\hbox {M}_{2}$ tides. The real (left column) and the imaginary (right column) parts of the three magnetic field components at the Earth’s surface calculated using the elmgFD code with 2-D surface conductance and forcing at the highest lateral resolution, $j_{\mathrm{max}}=480$. Note the use of different colour scales for vertical and horizontal components

Figure 4 shows the $\hbox {M}_{2}$ signatures predicted by the elmgFD code at the highest resolution of spherical harmonic degree 480. The magnetic field components are in general agreement with previous studies. We will use this figure as a reference to demonstrate the various effects with difference plots.

**Fig. 5:**
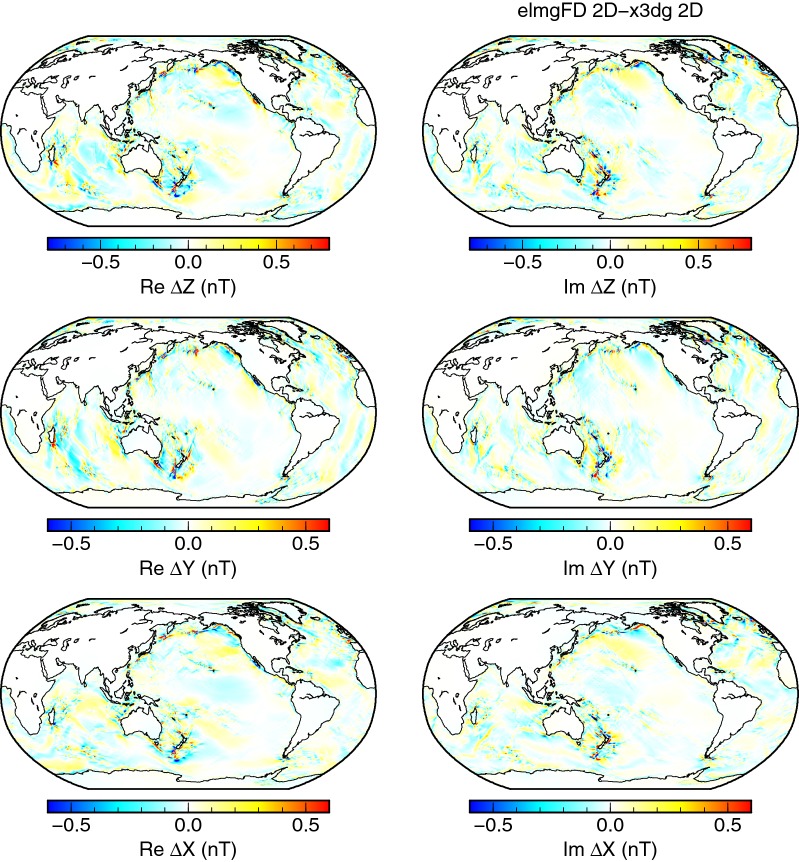
Differences between elmgFD and x3dg codes. Differences between the 2-D solutions calculated by the respective codes at the Earth’s surface for the highest resolution, $j_{\mathrm{max}}=480$ and $0.25^{\circ }\times 0.25^{\circ }$, respectively. Note that the colour scales in Figs. 5, 6, 7 and 9, 10 are reduced with respect to Fig. 4

First such difference is plotted in Fig. 5 where the 2-D solutions of elmgFD and x3dg at their respective highest resolutions are compared. As can be expected, the largest differences in all three components are concentrated in the areas where spatially complex tidal flows interact with strong vertical main magnetic field, such as in the Tasman sea and further south and east of New Zealand. Even there, the differences stay below 10% of the total predicted signal.

**Fig. 6:**
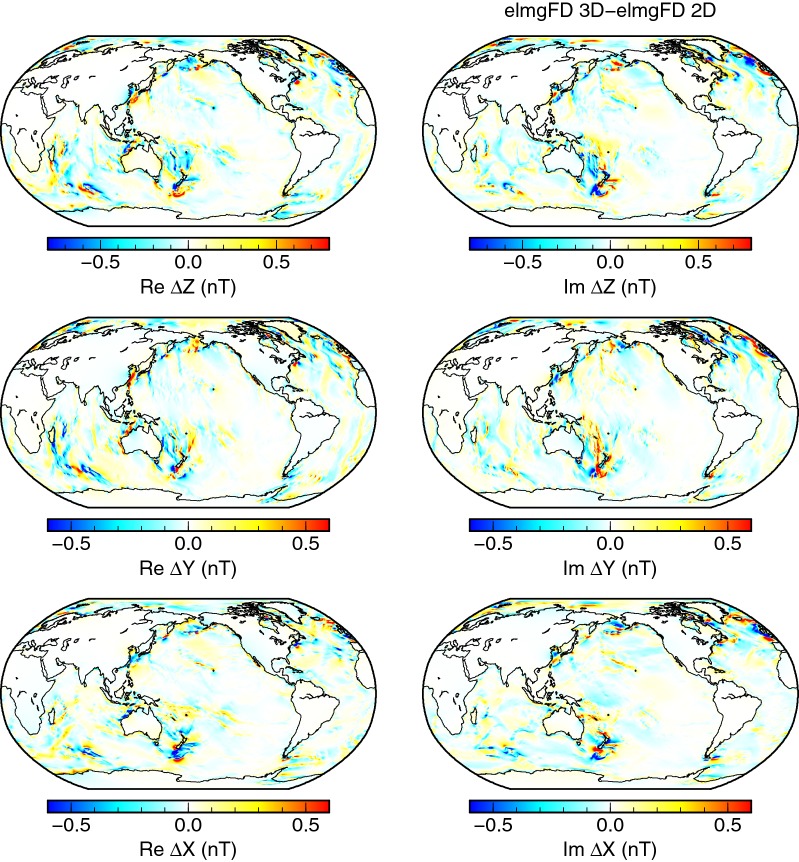
Effect of dimensionality. Differences between the 3-D and 2-D solutions calculated at the Earth’s surface with the elmgFD code at $j_{\mathrm{max}}=480$. The 3-D solution discretizes the ocean with 40 equidistant layers

**Fig. 7:**
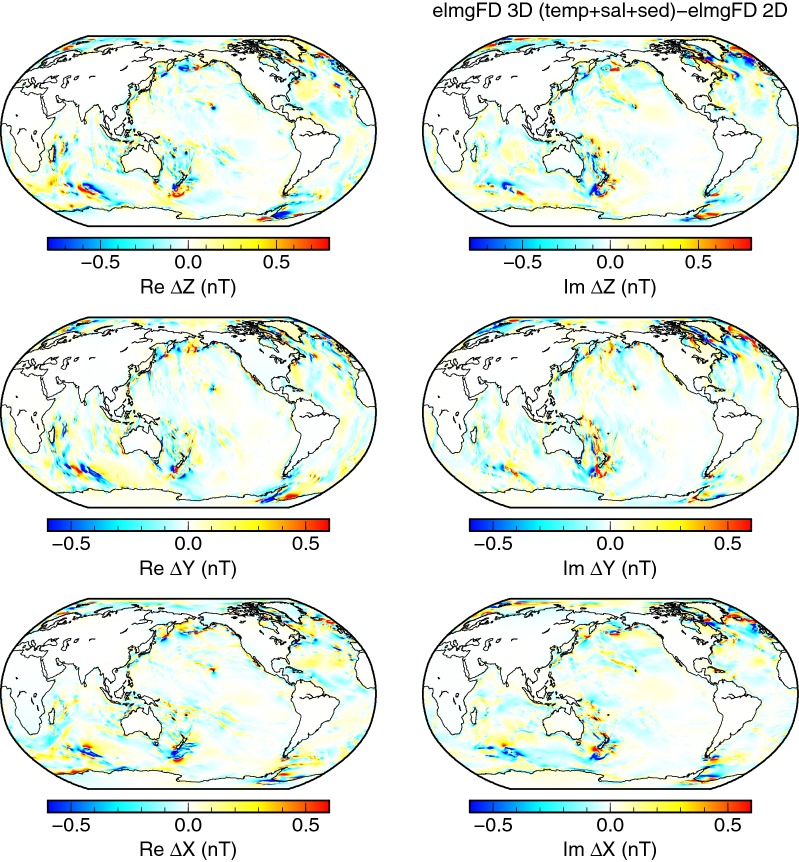
Effect of variable seawater conductivity and sediments. Differences between the 3-D and 2-D solutions calculated at the Earth’s surface with the elmgFD code at $j_{\mathrm{max}}=480$. The 3-D solution includes temperature and salinity-dependent seawater conductivity and variable sediment thickness and discretizes the ocean with 102 layers

The effect of dimensionality is shown in the difference plots in Figs. 6 and 7. The 3-D elmgFD solutions differ from the 2-D solution obtained by the same code mostly in the shallow areas, such as the Kerguelen plateau in the southern Indian ocean, the Bering sea, or the Rockall plateau in the northern Atlantic. In the more complicated $\sigma _{\mathrm{3D}}^{\mathrm {(T,s)}}$ model, the differences are slightly amplified, and detailed patterns of magnetic fields are modified, e.g. in the Weddell Sea, the Tasman Sea, and in the northern Atlantic. In these areas, the prediction of the 2-D model can be of even by 50% of the signal amplitudes. In view of these results, the 2-D approximation seems to be acceptable over deep oceans, where the differences are only slightly larger than those related to the choice of the modelling code.

### Effect of conductive mantle on the tidal signatures

**Fig. 8:**
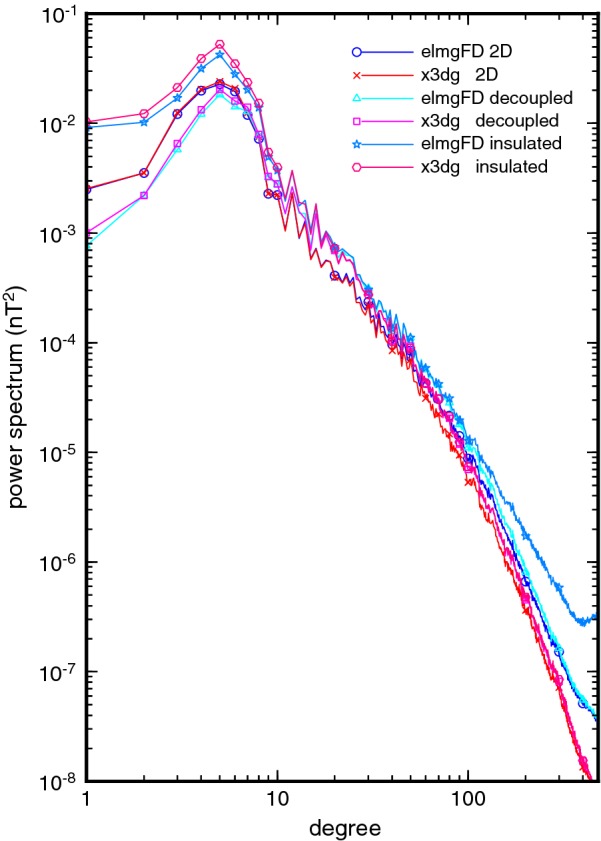
Effect of physical approximations on the geomagnetic power spectra. Geomagnetic power spectra calculated at the Earth surface by the 2-D approaches elmgFD and x3dg, and the respective effects of galvanically decoupled and insulated mantle. The lateral resolution was kept at $j_{\mathrm{max}}=480$ and $0.25^{\circ }\times 0.25^{\circ }$, respectively

**Fig. 9:**
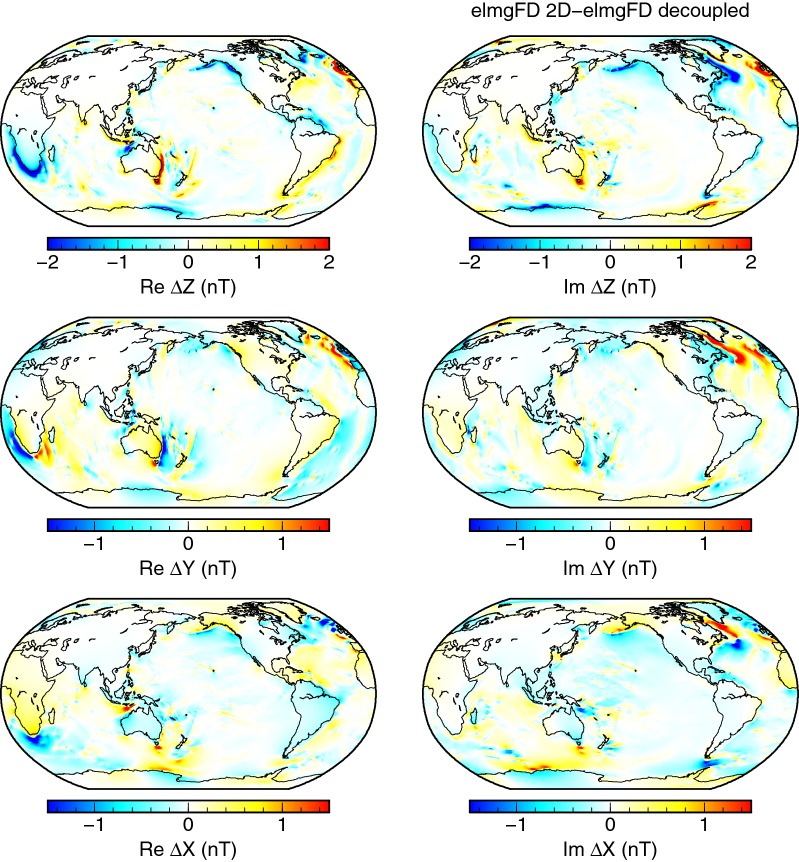
Effect of galvanically decoupled mantle. Differences between the 2-D solution and a unimodal solution without toroidal magnetic field, i.e. galvanically decoupled from the underlying conductive mantle. Both solutions were calculated at the Earth’s surface with the elmgFD code at $j_{\mathrm{max}}=480$

The effects of galvanic *decoupling* of the mantle and the *insulating* mantle are shown in Fig. 8 by means of power spectra at the Earth’s surface. In spite of implementation differences in both methods, the behaviour is consistent. For the *decoupled* model, the power spectra are reduced for degrees one to six and increased from degree seven onwards. This is in agreement with the theoretical arguments in Tyler (2017, Section 5.5). The spatial structure of the differences between the full 2-D model and the *decoupled* model is shown in Fig. 9. It is concentrated into the coastal areas, with the largest differences pronounced around southern Africa, on the Australian eastern coast, in the Labrador Basin, and west of the British Isles.

**Fig. 10:**
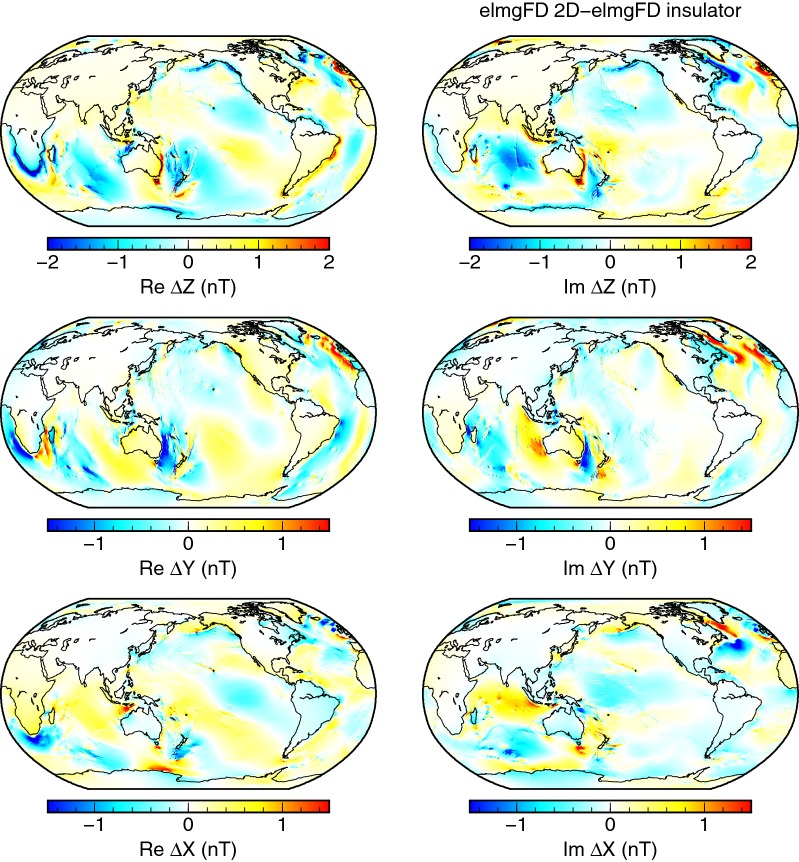
Effect of insulating mantle. Differences between the 2-D solution and a solution with perfectly insulating bottom boundary condition. Both solutions were calculated at the Earth’s surface with the elmgFD code at $j_{\mathrm{max}}=480$

In the case of *insulating* mantle, the effects become even more interesting. Judging from the spectra in Fig. 8, the amplitude of the signal is increased, especially at the lower degrees. The spatial pattern of differences, as displayed in Fig. 10, repeats most of the features from Fig. 9. However, the magnetic field is significantly strengthened over the deep oceans, missing the counteracting field generated by the large-scale induced currents in the mantle. This effect is evidently stronger than the galvanic coupling, shifting the entire spectrum upwards.

**Fig. 11:**
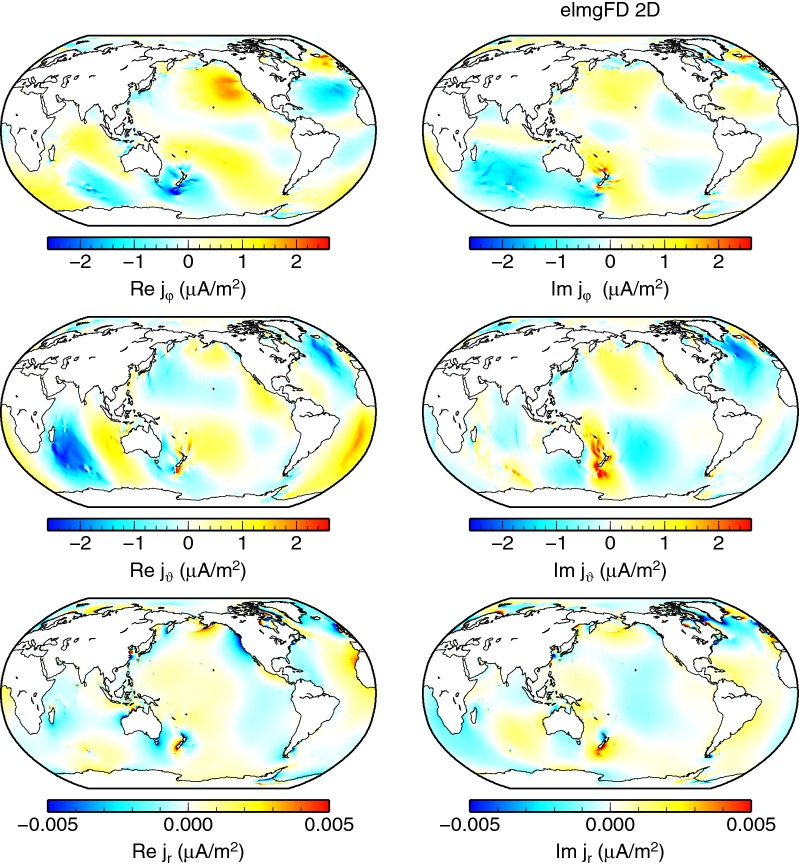
Electric current density at the seafloor. The eastward, southward, and radial components of the 2-D elmgFD solution at $j_{\mathrm{max}}=480$ are shown, respectively, from top to bottom

**Fig. 12:**
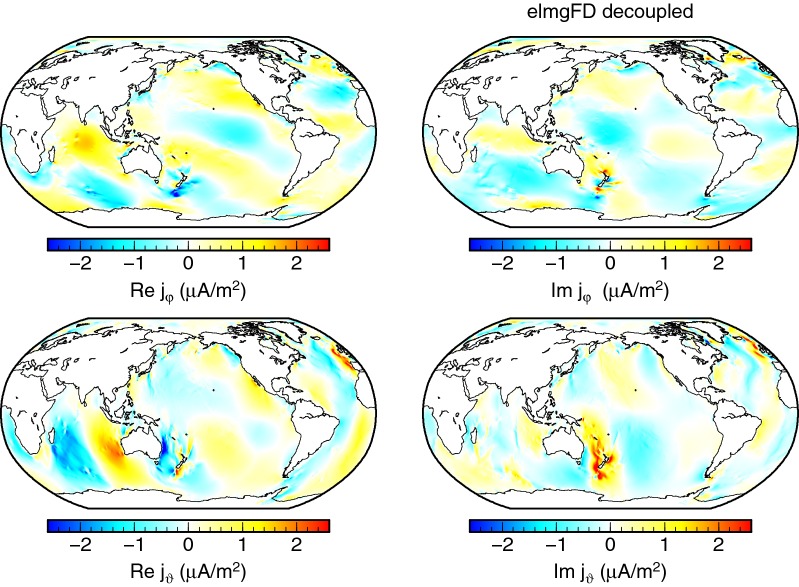
Electric current density at the seafloor. The horizontal components obtained for the *decoupled* solution. The radial component is zero everywhere in this case

**Fig. 13:**
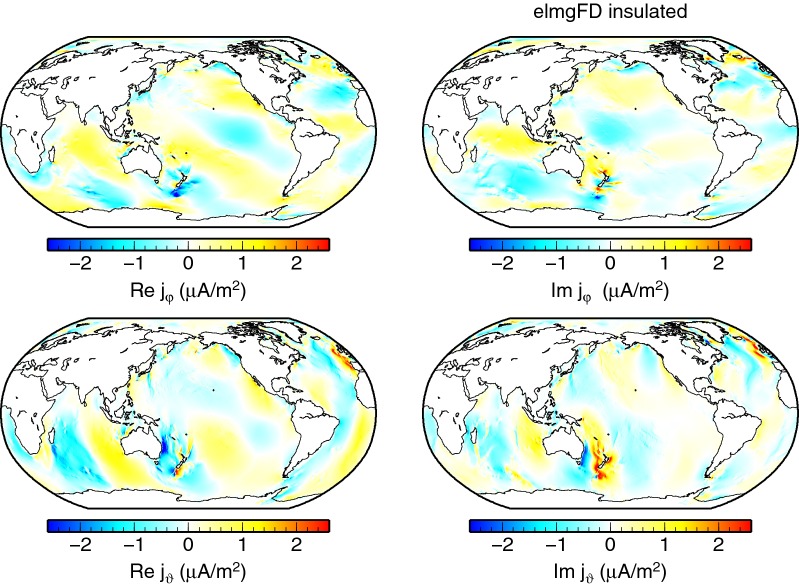
Electric current density at the seafloor. The horizontal components obtained for the *insulated* solution. The radial component is zero everywhere in this case

Another view on the full 2-D, the *decoupled*, and the *insulated* solutions is provided by means of electric current density at the seafloor, as plotted, respectively, in Figs. 11, 12 and 13. The vertical currents, which are present only in the full solution, are concentrated in the coastal areas. The pattern of the horizontal electric currents is changed and the amplitude mostly weakened in both the *decoupled* and *insulated* solutions.

## Conclusions

The recent advances in 3-D EM modelling allow us to calculate the magnetic signatures of the ocean tides in high resolution and in the full complexity. However, various physical approximations still can provide significant reduction of computational time and/or memory requirements, which can be exploited, especially in the inversion scenarios. We have evaluated the effect of various traditional approximations. The predictions of the 2-D approach match well with the 3-D approach locally above deep oceans, or in the interpretation of low-orbit satellite data dealing with spherical harmonic degrees below 20. However, for accurate prediction of signals at the coastal and island geomagnetic observatories, or more generally, in the areas with significant variations of bathymetry and coastlines, full 3-D calculations still may be required. This conclusion does not confirm the argument presented by Tyler (2017, Section 5.2) that the Earth oceans behave as an electrically thin sheet for periods above 10 min. Note that Tyler’s criterion is based on the penetration depth of magnetic field from the boundary. However, the imposed electric currents are distributed everywhere in the ocean volume and for tidal movements do not decrease significantly with depth.

The 3-D approach presented here is still based on the 2-D barotropic tidal flows. The prediction of the magnetic field induced by the 3-D tidal flows comprising the baroclinic component (Stammer et al. 2014) has not yet been performed. Although the comparison of Saynisch et al. (2018) includes several baroclinic models, the induced magnetic field was still calculated from horizontal transports only.

The electromagnetic interactions of the oceans with the underlying conductive mantle are significant enough to be treated comprehensively in numerical modelling, including the galvanic coupling and possibility of vertical electric currents flowing from or into the mantle. The omission of the galvanic coupling leads to an underestimation of the tidal signals at degrees below six, in agreement with the analytical estimates by Tyler (2017, Section 5.5). Using a completely insulating mantle has the opposite effect.
